# On the Child’s Right to Bodily Integrity: When Is the Right Infringed?

**DOI:** 10.1093/jmp/jhab013

**Published:** 2021-07-05

**Authors:** Joseph Mazor

**Affiliations:** Duke Kunshan University, Kunshan, China

**Keywords:** best interests, bodily integrity, children’s rights

## Abstract

This article considers two competing types of conceptions of the pre-autonomous child’s right to bodily integrity. The first, which I call encroachment conceptions, holds that any physically serious bodily encroachment infringes on the child’s right to bodily integrity. The second, which I call best-interests conceptions, holds that the child’s right to bodily integrity is infringed just in case the child is subjected to a bodily encroachment that substantially deviates from what is in the child’s best interests. I argue in this article that best-interests conceptions are more plausible than encroachment conceptions. They have more attractive implications regarding the permissibility of interventions in children’s bodies that are beneficial for the child but are not medically necessary. They are better able to explain the moral distinction between cases in which an encroachment on a child’s body is needed to benefit that child and cases in which an encroachment on one child’s body is needed to benefit another. Finally, best-interests conceptions are more consonant than encroachment conceptions with our understanding of adults’ right to bodily integrity.

## I. INTRODUCTION

In many debates about interventions in young children’s bodies (e.g., male infant circumcision), opponents of the practice claim that violations of the child’s right to bodily integrity are occurring (e.g., Denniston, 1999), whereas proponents of the interventions deny this claim (e.g., Benatar and Benatar, 2003). These disputes are often based, not only on empirical disagreements, but also on conceptual disagreements regarding the child’s right to bodily integrity, and in particular, the conditions under which this right is infringed. This is the issue I wish to consider in this article.

There are two broad ways of understanding the infringement conditions for the child’s right to bodily integrity. Some scholars hold that the child’s right to bodily integrity is infringed whenever there is a *physically serious* encroachment on the child’s body (e.g., Rahman and Toubia, 2000, 3). Other scholars hold that the child’s right to bodily integrity is infringed just in case the child is subjected to a bodily intervention that substantially deviates from what is *in her best interests* (Vallentyne, 2002, 994). Scholars in both camps generally assert their preferred position without defending it and often fail to even acknowledge the competing view.^1^

However, this issue is not some minor conceptual disagreement. As I demonstrate below, our understanding of the child’s right to bodily integrity can influence our view of the permissibility of interventions in children’s bodies in important ways. There is thus substantial value in attempting to resolve this conceptual controversy.

In this article, I argue that understanding the child’s right to bodily integrity in terms of her best interests rather than in terms of the physical seriousness of the encroachment is more plausible for several reasons. First, when the two conceptions generate clearly diverging implications, the implications of the best-interests conceptions are more plausible. Second, best-interests conceptions can contend with both intrapersonal and interpersonal moral dilemmas more straightforwardly than can encroachment conceptions. Finally, best-interests conceptions cohere better with commonly accepted understandings of adults’ right to bodily integrity.

## II. THE SCOPE OF THE INQUIRY

Let me begin by clarifying the scope of my inquiry. First, I am interested here in the child’s *moral* right to bodily integrity rather than the legal right to bodily integrity. Although there are important connections between moral and legal rights, there are often special considerations in the case of legal rights (e.g., costs of enforcement, coherence with constitutions) that make it useful to consider the two types of rights separately.

Second, the interventions that I wish to consider here are those authorized by the child’s parents (or legal guardians more broadly). I leave open the possibility that the child’s right against bodily encroachments is defined differently in the case of encroachments by strangers.

Finally, I assume that the children in question are *pre-autonomous*. That is, they are not yet able to grant or withhold morally meaningful consent. There is an important debate in the children’s rights literature regarding when precisely children’s consent is worthy of moral consideration and regarding what role this consent should play (Alderson, 1993). I set these complex issues aside by considering the right to bodily integrity of children too mentally immature to count as even partially autonomous.^2^

Thus, the question I consider here is the conditions under which the (pre-autonomous) child’s (moral) right to bodily integrity is infringed (with respect to interventions authorized by the child’s legal guardians). However, for expositional simplicity, I exclude the modifiers in parentheses in the rest of this article.

## III. TWO WAYS TO UNDERSTAND A CHILD’S RIGHT TO BODILY INTEGRITY

The different ways of understanding the child’s right to bodily integrity can perhaps be best illustrated by examining how they apply to a concrete case. Consider the following example introduced by Eliyahu Ungar-Sargon:

**Life-Saving Amputation**: A child is injured in a car accident, and the only way to save his life is to amputate his limb. The child’s parents authorize the amputation. (2015, 186)

Have the parents infringed on the child’s right to bodily integrity in this case?

### Encroachment Conceptions

Scholars who endorse *encroachment conceptions* (as I call them) of the child’s right to bodily integrity would generally give an affirmative answer to this question. Encroachment conceptions hold that an intervention in the body of a child constitutes an infringement of her right to bodily integrity just in case it constitutes a *physically serious bodily encroachment*. Different encroachment conceptions appeal to different understandings of “physically serious,” with some focusing on substantial alteration, irreversibility, damage to (or removal of) healthy tissue, loss of bodily function, and/or some other criterion.^3^ However, the defining feature of encroachment conceptions is that “physical seriousness” is *not* defined in terms of the effects on the child’s interests. Since the amputation of the limb is a physically serious encroachment on the child’s body,^4^ it constitutes an infringement of the child’s right to bodily integrity on this view.

A variety of thinkers endorses encroachment conceptions. For example, Merkel and Putzke write:

[Consider] the child’s bodily integrity. There we do have a distinct and definite criterion with which to identify the outer boundary of parental authority: simply the bounds of the child’s skin . . . Any substantial and permanent lesion upon the physical *gestalt* of a child is rightly considered an unjustified harm.^5^ (2013, 444–5)

Rahman and Toubia also appeal to an encroachment conception when they write,

[T]he cutting of healthy genital organs for non-medical reasons is at its essence a basic violation of girls’ and women’s right to physical integrity. This is true regardless of the degree of cutting or the extent of the complications that may or may not ensue. (2000, 3)

Importantly, though proponents of encroachment conceptions view Life-Saving Amputation as an infringement of the child’s right to bodily integrity, they need not view it as impermissible. As Ungar-Sargon (2015) argues, although the amputation infringes on the child’s right to bodily integrity, the amputation is permissible *all things considered* because the child’s right to life, which requires that the amputation be done, outweighs the importance of respecting the child’s right to bodily integrity in this case. Indeed, all proponents of encroachment conceptions recognize that infringements of the child’s right to bodily integrity are permissible in cases of medical necessity.

### Best-Interests Conceptions

There is, however, a different way of conceptualizing the child’s right to bodily integrity—one that denies that an infringement of the child’s right to bodily integrity occurs in Life-Saving Amputation. *Best-interests conceptions* (as I call them) hold that an intervention in the child’s body infringes on the child’s right to bodily integrity just in case the intervention substantially deviates from what is in the child’s best interests.^6^ Proponents of best-interests conceptions recognize that Life-Saving Amputation constitutes a very serious encroachment on the child’s body. They also recognize that the child has *interests* in keeping the limb and in avoiding the traumatic operation needed to amputate it, and that these interests must be taken into account when determining whether the amputation is in his best interests overall. However, since the amputation is clearly in the child’s best interests all things considered, his right to bodily integrity is not infringed in this case, according to best-interests conceptions.

Several scholars support best-interests conceptions. For example, Peter Vallentyne writes, “Consider a child’s interest-protecting right of personal security . . . It does not rule out contact when the child’s interests are not adversely affected . . . Agents are permitted to impose short-term bodily harms on children—as long as the net long-term effects are suitably nonharmful” (2002, 994). Describing the American Supreme Court’s approach to the child’s right to bodily integrity, B. Jessie Hill writes, “For younger children . . . the bodily integrity right . . . is essentially a right of children to have their best interests protected” (2015, 1316).

Different best-interests conceptions endorse different positions about how much leeway parents should be given in determining what is in their child’s best interests in cases in which the parents and the state disagree.^7^ Different conceptions also have diverging accounts of the perspective from which the child’s interests should be judged. One possibility, known as the “substituted judgment standard,” is that the right standard involves ascertaining what *the particular child herself* would choose once she is an adult (Svoboda, 2013, 470). Another possibility is that the child’s interests should be judged by asking what a “rational person” would want in a similar situation (Svoboda, Van Howe, and Dwyer, 2000, 77, 88). However, for the purposes of this article, I classify both of these approaches as best-interests conceptions, since they both define infringements on the child’s right to bodily integrity in terms of the child’s well-being rather than in terms of the physical seriousness of the encroachment.

## IV. THE IMPORTANCE OF THE CONTROVERSY

Resolving the controversy between best-interests conceptions and encroachment conceptions is important, not only for the sake of conceptual clarity, but also because of the controversy’s implications for the permissibility of interventions in children’s bodies. Admittedly, in the example of Life-Saving Amputation, both types of conceptions hold that the amputation is permissible (albeit for different reasons). However, in other cases, encroachment conceptions and best-interests conceptions can lead to diverging judgments regarding the permissibility of interventions in children’s bodies.

Consider, for example, the case of male infant circumcision. If we accept a best-interests conception of the child’s right to bodily integrity, then we will be inclined to permit circumcision as long as it is in the child’s best interests. Considerations such as minor health benefits (Benatar and Benatar, 2003) and, for children in majority-circumcised communities, the potential psychosocial benefits of not being atypical, might be seen as sufficient to make the practice in the child’s best interests and thus not an infringement of his right to bodily integrity.

However, if we accept the encroachment conception, our judgment in an identical case with identical empirical assumptions may be different. Since circumcision constitutes a serious encroachment on the child’s body (on a variety of conceptions of what “serious” means), it is an infringement of the child’s *right* to bodily integrity. Assuming that the child’s right to bodily integrity should be granted substantial weight, the relatively minor health and psychosocial benefits assumed above may no longer be sufficient to justify the procedure.

More generally, encroachment conceptions set *a substantially higher bar* that the benefits a physically serious encroachment on a child’s body must meet before the encroachment can be justified (since its benefits must justify a rights-infringement). Just how large the benefits for the child have to be will depend on the strength attributed to the child’s right to bodily integrity.^8^ Proponents of encroachment conceptions who assign the child’s right to bodily integrity a great deal of weight might even insist that serious encroachments on the child’s body can only be justified in the case of *medical necessity*.^9^ This is a much more stringent requirement than proponents of the best-interests conceptions would endorse.

## V. BENEFICIAL BUT MEDICALLY UNNECESSARY SERIOUS ENCROACHMENTS

The divergence in the different conceptions’ implications regarding the permissibility of interventions in children’s bodies not only underlines the importance of this controversy. It also suggests a path toward resolving it. If we can find interventions whose permissibility status seems intuitively clear, but where the two types of conceptions come to different conclusions, this could help settle the debate.

Admittedly, finding such interventions is no easy matter. Many of the serious encroachments on children’s bodies that are uncontroversial are also medically necessary. Both types of conceptions agree that such interventions are permissible. Moreover, since any physically serious encroachment on the child’s body will substantially set back some of the child’s important interests (e.g., in avoiding pain, in avoiding loss of functional tissue, etc.), serious physical encroachments with very minor benefits for the child (and no other considerations in their favor) will generally be rejected by both types of conceptions as impermissible infringements on the child’s right to bodily integrity. What is needed are cases of physically serious encroachments whose permissibility status is fairly clear and whose benefits are substantial but not of the weightiest kind.

One example that arguably falls into this category is the following case:

**Cleft Lip Operation**: A child is born with a minor cleft lip.

Assume:

• The cleft lip is sufficiently minor so that there is no medical reason for performing the operation. The physical function of the mouth would be unaffected by the cleft and the cleft’s psychosocial consequences are not anticipated to lead to mental health problems or to severe social exclusion for the child.

• It is significantly more costly for the child in various ways (more painful, more dangerous, longer recovery period, greater chance of permanent scarring, highly probable moderate teasing as a child) to wait until the child is autonomous to carry out the operation.^10^

• The operation requires general anesthesia (including the associated miniscule risk of death), removing at least some healthy tissue, and modestly reduce the flexibility of a part of the surrounding area of the face in order to preserve the typical “cupid’s bow” curvature of the lips.^11^

• The operation is irreversible. Society does not have the medical knowledge to reintroduce a cleft lip in adults, at least not without substantially compromising facial movement in the surrounding facial area.

• The parents decide to authorize the operation.

I take it that the permissibility status of this procedure is fairly clear (it is permissible). This is also a procedure with benefits for the child that are substantial, yet *ex hypothesi* not of the weightiest kind. It is thus a good case for evaluating the plausibility of the different conceptions.

If this procedure is permissible, this lends support to best-interests conceptions. These conceptions can straightforwardly explain the permissibility of this procedure. After all, the operation seems clearly in the best interests of the child. While the psychosocial and esthetic benefits of the operation do not rise to the level of medical necessity, they do seem to clearly outweigh the operation’s risks and costs. Thus, according to best-interests conceptions, Cleft Lip Operation does not infringe on the child’s right to bodily integrity.

Proponents of encroachment conceptions, on the other hand, face substantial difficulty explaining the permissibility of Cleft Lip Operation. This operation causes an irreversible alteration of the body that entails some risk of death, requires the removal of healthy tissue, and negatively affects the functioning of the surrounding facial area. It is thus a physically serious encroachment on the child’s body (on a variety of conceptions of what “physically serious” entails) and therefore constitutes an infringement of the child’s right to bodily integrity according to encroachment conceptions. Moreover, while the operation’s esthetic and psychosocial benefits for the child are certainly substantial, it is unclear that they are sufficiently weighty to justify something as serious as an infringement of *a right*. Certainly, these countervailing benefits do rise to the level of medical necessity. It is thus unclear whether proponents of encroachment conceptions who also view the child’s right to bodily integrity to be very weighty have the theoretical resources to explain the permissibility of this procedure.

Proponents of encroachment conceptions might respond by endorsing a *weaker* right to bodily integrity in this case—one whose infringement would be justified by the substantial esthetic and psychosocial benefits to the child of correcting the minor cleft. However, even these less extreme encroachment conceptions face an important intuitive difficulty. Namely, they seem committed to viewing Cleft Lip Operation as a *hard* case—one that confronts us with powerful competing moral considerations (an infringement of the child’s right to bodily integrity versus weighty benefits for the child). Yet Cleft Lip Operation is, I submit, *fairly clearly* permissible. The kind of powerful, intuitive pull in both directions that one would expect in the context of a genuine moral dilemma seems absent in this case.

Best-interests conceptions do not face this intuitive difficulty. While important interests are indeed set back by the operation (e.g., the interests in avoiding pain and some loss of function), the operation is, I submit, *fairly clearly* in the best interests of the child overall. It is thus clearly *not* an infringement of the child’s right to bodily integrity according to best-interests conceptions and therefore does not fall in the category of a hard case.

Proponents of encroachment conceptions might concede that best-interests conceptions face less difficulty in explaining the permissibility of Cleft Lip Operation. However, they might appeal to other instances of physically serious encroachments on children’s bodies in which encroachment conceptions ground more plausible judgments than do best-interests conceptions. Examples include:

- Child ear piercing for baby girls,
- Height growth hormonal treatments for boys of slightly below average height, and
- Double eyelid surgery for an East Asian child done to avoid racism in a Caucasian community.^12^

Since these interventions are morally problematic (if not impermissible), and since they appear to be in the best interests of the child (or at least could be on plausible further specification of the cases), these examples pose a serious challenge to best-interests conceptions.

However, proponents of best-interests conceptions have the theoretical resources to explain the problematic nature of these interventions. Consider first the ear piercing for baby girls. At first glance, it might seem as though this procedure is in the best interests of the girl. After all, in many societies, a substantial majority of women eventually choose to have their ears pierced. Moreover, the baby, unlike older girls, does not experience the anticipatory dread of the procedure. Finally, the parents can arguably more easily ensure that the baby’s ears are properly cared for compared with waiting until the child is sufficiently autonomous to give meaningful consent for the procedure.

Nevertheless, proponents of best-interests conceptions can explain the problematic nature of this intervention by appealing to the girl’s *weighty interest in future bodily autonomy*—in being able to decide for herself once she is autonomous what is to be done with her body.^13^ Indeed, by recognizing this interest, proponents of best-interests conceptions can condemn (or at least recognize the difficulty of endorsing the permissibility of) any serious encroachment whose costs of delay for the child are minimal.

Note, however, that recognizing the child’s interest in future bodily autonomy need not change best-interests conceptions’ verdict in Cleft Lip Operation for two reasons. First, we might plausibly view the strength of a child’s interest in future bodily autonomy as depending on how likely she is to reflectively reject her parents’ decision. While many girls might regret having had their ears pierced (e.g., due to rejecting gender norms), a much smaller percentage of children who have their cleft lip corrected are likely to regret having been subjected to this encroachment as infants. Second, the costs of delay in Cleft Lip Operation (increased medical complexity of the procedure and likely teasing as a child) are much more substantial than in the ear-piercing case and can therefore more plausibly outweigh the child’s interest in future bodily autonomy. Thus, proponents of best-interests conceptions can explain the problematic nature of ear piercing for baby girls by appealing to the interest in future bodily autonomy while affirming the relatively unproblematic nature of Cleft Lip Operation.

Proponents of best-interests conceptions also have the theoretical resources to condemn the height growth hormonal treatments for boys of slightly below average height. Admittedly, this intervention may be in each affected boy’s best interests, even taking into account his interest in future bodily autonomy.^14^ However, proponents of best-interests conceptions can appeal to values besides the child’s right to bodily integrity to explain the problematic nature of this intervention. For example, as several theorists have argued, there are good moral reasons to insist that parents not enter a kind of “arms race” of physical enhancements for their children (Sandel, 2004), even if these enhancements are in an individual child’s best interests. By appealing to the other values set back by such an arms race, proponents of best-interests conceptions can condemn the growth hormone treatment while nevertheless affirming that it does not infringe on the child’s right to bodily integrity.

Note that proponents of best-interests conceptions can recognize the problem with the growth hormone treatment while still affirming the unproblematic nature of Cleft Lip Operation. After all, while administering growth hormones to boys can change the social understanding of “typical” height and thus can launch an “arms race” of parents who wish to ensure that their sons are not atypically short, permitting a correction of a cleft lip does not alter the conception of a “typical” lip shape and thus is much less likely to launch a morally problematic “arms race.”

Finally, proponents of best-interests conceptions can explain the problematic nature of the child double-eyelid surgery done to accommodate racist preferences through a combination of the two strategies highlighted above. First, they can question whether this procedure really is in the best interests of the child by appealing to the child’s interest in future bodily autonomy. The child might, after all, plausibly grow up to prefer being treated in a discriminatory manner to being physically reshaped due to others’ racist preferences. Second, proponents of best-interests conceptions can once again appeal to values besides the child’s right to bodily integrity to explain the problematic nature of this procedure. As several thinkers have argued, there may be serious moral problems with shaping children to accord with racist norms (Aquino, 2017).

Note that, once again, best-interests conception proponents can recognize the problematic nature of the double eyelid surgery while still affirming the relatively unproblematic nature of Cleft Lip Operation. It is true that the parents who authorize Cleft Lip Operation are shaping their children to fit esthetic norms. However, there is a variety of reasons why acceding to racist norms is more problematic than acceding to esthetic norms. For one thing, in acceding to the racist norms, the parents might inadvertently lead the child to view *a key part* of *her identity* as pathological or inherently inferior (Aquino, 2017). A correction of a cleft lip, on the other hand, while certainly expressing a view about the undesirability of certain physical features, is far less likely to lead the child to question the worth of core aspects of her identity.

Thus, proponents of best-interests conceptions have plausible strategies for explaining both the unproblematic nature of Cleft Lip Operation and the problematic nature of interventions such as ear piercing for baby girls, hormone growth treatment for boys of slightly below average height, and double eyelid surgery done to mitigate racist discrimination against an Asian child. On the other hand, while proponents of encroachment conceptions can explain the problematic nature of these three encroachments on children’s bodies, they face substantial difficulties in explaining the permissibility of Cleft Lip Operation.

## VI. INTRAPERSONAL/INTERPERSONAL CONSISTENCY

There is also a second class of cases in which best-interests conceptions seem to ground more plausible judgments compared with encroachment conceptions: cases in which physically serious encroachments on the body of one child are needed to benefit another child.

Consider the following example, which is an interpersonal variation of Life-Saving Amputation considered above:

**Life-Saving Kidney Transfer**: Child A is injured in a car accident, and the only way to save his life is to perform an immediate kidney transplant. Child B is at the hospital for a routine check-up and is the only available match for Child A.

Child B’s parents authorize the kidney transplant.

I take it that Child B’s parents have infringed on Child B’s right to bodily integrity and that the operation is impermissible.^15^ Although the aggregate benefits of the parents’ actions are in some sense positive, the action nevertheless seems like an unacceptable violation of Child B’s rights.

Best-interests conceptions are able to straightforwardly contend with this case. In the *intrapersonal* Life-Saving Amputation, best-interests conceptions imply that the child’s right to bodily integrity is not infringed because the intervention is clearly in the child’s best interests. However, in the *interpersonal* Life-Saving Kidney Transfer, Child B’s right to bodily integrity *is* infringed, because the removal of her body part substantially deviates from what is in *her* best interests. If we further insist (not implausibly) that a child’s right to bodily integrity (a negative right against interference) is stronger than the right a child has to the organs needed to survive (a positive right to assistance),^16^ then we can straightforwardly explain why the encroachment in the intrapersonal Life-Saving Amputation is permissible while the encroachment in the interpersonal Life-Saving Kidney Transplant is not.

Proponents of encroachment conceptions, on the other hand, face substantially greater difficulty in explaining both cases in a consistent way. After all, according to encroachment conceptions, in both cases, we have an infringement of one child’s right to bodily integrity on the one hand and respect for one child’s right to life on the other. If a child’s right to life outweighs the infringement of a child’s right to bodily integrity in Life-Saving Amputation, then it is unclear why the respect for Child A’s right to life is insufficient to justify an infringement of Child B’s right to bodily integrity.

Admittedly, proponents of encroachment conceptions can pursue a variety of strategies to address this problem. They might, for example, insist on a special weighting when rights are traded off intrapersonally as opposed to interpersonally. However, the capacity of best-interests conceptions to plausibly contend with both types of cases without any additional philosophical machinery is another consideration in their favor.

## VII. COHERENCE WITH THE ADULT’S RIGHT TO BODILY INTEGRITY

A final consideration in favor of best-interests conceptions is that they are more consonant with generally accepted understandings of adults’ right to bodily integrity compared with encroachment conceptions. Of course, the pre-autonomous child’s right to bodily integrity is different from the adult’s right to bodily integrity in important ways. However, a theoretical coherence between a particular conception of the child’s right to bodily integrity and accepted conceptions of the adult’s right to bodily integrity makes the case for that conception substantially stronger.

Note first that, in the case of adults, informed consent is fundamental to the right to bodily integrity, while physical seriousness (in the sense used by the proponents of encroachment conceptions) plays, at most, a minor role (Svoboda, Van Howe, and Dwyer, 2000, 63–4). A doctor who performs a life-saving amputation of a limb of an adult who has provided informed consent cannot be plausibly accused of infringing the adult’s right to bodily integrity, even though this is clearly a serious encroachment on the adult’s body. Moreover, even nonserious encroachments on an adult’s body without informed consent can infringe the right to bodily integrity. For example, although cutting a very young child’s hair is not generally seen as a serious encroachment on her body, I take it that cutting an adult’s hair without her permission would constitute an infringement of her right to bodily integrity.^17^

Since what fundamentally matters in the case of autonomous adults is not the physical seriousness of the encroachment but rather the presence or absence of informed consent, the focus on physical seriousness in the case of children is puzzling. It seems much more consonant with our understanding of the adult’s right to bodily integrity to define the child’s right to bodily integrity in terms of some appropriate *analog* to the autonomous adult’s informed consent.

An obvious candidate for such an analog is some type of best-interests standard. After all, it is widely recognized that one of the key moral roles that informed consent plays in the case of adults is as an indication of what is in the adult’s best interests (e.g., Zwolinski, 2007, 693–5). Moreover, in cases in which obtaining informed consent from an adult is impossible, it is broadly accepted that physicians should intervene in adults’ bodies on the basis of what is in the adult’s best interests, as would be judged by the adult herself, were she able to make the appropriate decision (e.g., Svoboda, Van Howe, and Dwyer, 2000, 75–9). Since an adult’s right to bodily integrity is foundationally defined in terms of informed consent rather than the physical seriousness of the bodily encroachment, and since the best-interests standard is a plausible analog to informed consent in cases where such consent is impossible to obtain, best-interests conceptions of the child’s right to bodily integrity are more consonant with accepted conceptions of adults’ right to bodily integrity and are for this reason, too, more plausible.

## VIII. CONCLUSION

The proper conceptualization of the child’s right to bodily integrity is an important unresolved issue in the ethics of interventions in children’s bodies. I have argued in this article that conceptions of this right that focus on the child’s best interests are more plausible than conceptions that focus on the physical seriousness of the bodily encroachment. In cases in which the two conceptions have clearly diverging implications about permissibility, the implications of best-interests conceptions seem more plausible. Moreover, unlike encroachment conceptions, best-interests conceptions can straightforwardly explain the difference between cases in which a serious encroachment is needed to benefit the same child and cases in which a serious encroachment on the body of one child is needed to benefit another. Finally, best-interests conceptions of the child’s right to bodily integrity cohere better with generally accepted understandings of the adult’s right to bodily integrity.

Best-interests conceptions imply that even moderate benefits for the child will sometimes be sufficient to justify a physically serious encroachment on that child’s body. They also imply that it is insufficient to simply point to some physically serious encroachment on the child’s body (e.g., a substantial, permanent alteration or removal of healthy tissue) to ground the claim that the child’s right to bodily integrity has been infringed. Instead, any such claim will have to be grounded in a careful analysis of whether and to what extent the encroachment in question is in the best interests of the child.

## Footnotes

1. For an important exception in which a competing view is considered, albeit briefly, see Benatar and Benatar (2003, 36).

2. However, the way in which we define the rights of pre-autonomous children to bodily integrity will almost surely have important implications for our understanding of this right for older children.

3. Some of the possible variations can be seen in the work of the encroachment conception proponents cited below.

4. Although part of the limb can be assumed to be irreparably damaged, we can also assume that there is no way to avoid removing substantial amounts of healthy tissue in the amputation.

5. Merkel and Putzke (2013, 445) go on to state that as long as the encroachment advances the child’s well-being, then the parents can validly authorize it. However, this later caveat, which is effectively an endorsement of a best-interests conception, is inconsistent with the quote cited above, since it implies that at least some substantial and permanent lesions on the physical *gestalt* of children *can* be justified, as long as they are in the child’s best interests.

6. To see why there needs to be a *substantial* deviation from the child’s interests, consider two operations that are very close to each other in terms of the child’s welfare, though one is *slightly* less welfare-promoting. Assume also that there are strong considerations (e.g., having to do with the more welfare-promoting operation’s expense and the effects of the resulting financial strain on the other siblings) in favor of the slightly less welfare-promoting operation. I submit that the child’s right to bodily integrity would not be infringed if the parents chose the slightly less welfare-promoting operation. I am grateful to Peter Vallentyne for this nuanced point.

7. For a discussion of the legal controversies over the limits of parental authority to determine what is in the child’s best interests, see Svoboda, Van Howe, and Dwyer (2000, 83–92).

8. Ungar-Sargon (2015, 186), for example, seems to endorse the view that the child’s right to bodily integrity is a trump (meaning that only other rights are sufficiently weighty to justify its infringement. Mere interests can never justify the infringement of this right). For a discussion of the right-as-trump view, see Dworkin (1984).

9. For an example of such a position, see Denniston, Hodges, and Milos (1999, vi). Although it is admittedly not clear that an encroachment conception of the child’s right to bodily integrity undergirds this position, it may well play a role.

10. As Dr. Gulli writes, “[o]ptimal results occur when the first operation is performed between two and six months of age” (Gulli et al., 2021).

11. For a description, see Gulli et al. (2021).

12. Ungar-Sargon (2015, 186) uses this example to defend his encroachment conception against a rival view that shares some similarities with the view I am defending here.

13. For a discussion of the importance of this interest in future bodily autonomy, see Earp (2015, 98–100).

14. After all, few boys are likely to regret being slightly taller than average rather than slightly shorter than average. And the intervention may well be such that it cannot be delayed until the child is fully autonomous.

15. For a review of the relevant law relating to such cases, see Murphy (1978). There are admittedly some cases in which parents have been allowed to authorize kidney transplants from one child to another in the case of siblings. However, a plausible case could be made that the child would herself have consented to make a limited sacrifice to save *her sibling* if she had the capacity to grant consent. Such an argument based on the child’s interests is far less plausible in the case of an unrelated child.

16. For a discussion of positive versus negative rights, see Wenar (2015, Sec. 2.1.8).

17. Indeed, judges in the United Kingdom agreed that cutting hair was a bodily harm. For a description of the case, see Leonard (2006).
